# Do We View Robots as We Do Ourselves? Examining Robotic Face Processing Using EEG

**DOI:** 10.3390/brainsci16010009

**Published:** 2025-12-22

**Authors:** Xaviera Pérez-Arenas, Álvaro A. Rivera-Rei, David Huepe, Vicente Soto

**Affiliations:** Center for Social and Cognitive Neuroscience, School of Psychology, Universidad Adolfo Ibáñez, Diagonal Las Torres 2640, Peñalolén 80305, Chile; xaarenas@alumnos.uai.cl (X.P.-A.); alvaro.rivera@uai.cl (Á.A.R.-R.); david.huepe@gmail.com (D.H.)

**Keywords:** electroencephalography, emotional facial expressions, event-related potentials, face processing, robotic faces, visual decoding

## Abstract

**Background/Objectives:** The ability to perceive and process emotional faces quickly and efficiently is essential for human social interactions. In recent years, humans have started to interact more regularly with robotic faces in the form of virtual or real-world robots. Neurophysiological research regarding how the brain decodes robotic faces relative to human ones is scarce and, as such, warrants further research to explore these mechanisms and their social implications. **Methods:** This study uses event-related potentials (ERPs) to examine the neural correlates during an emotional face categorization task involving human and robotic stimuli. We examined differences in brain activity elicited by viewing robotic and human faces expressing both happy and neutral emotions. ERP waveforms’ amplitudes for the P100, N170, P300, and P600 components were calculated and compared. Furthermore, mass univariate analysis of ERP waveforms was carried out to explore effects not limited to brain regions previously reported in the literature. **Results:** Results showed robotic faces evoked increased waveform amplitudes at early components (P100 and N170) as well as at the later P300 component. Further, only mid-latency and late cortical components (P300 and P600) showed amplitude differences resulting from emotional valences, aligning with dual-stage models of face processing. **Conclusions:** These results advance our understanding of face processing during human–robot interaction and contribute to our understanding of brain mechanisms underlying interactions when viewing social robots, setting new considerations for their use in brain health settings and broader cognitive impact.

## 1. Introduction

Human face processing is a hallmark feature of social cognition and an essential trait in human visual processing. The capacity to correctly detect and decode human faces has been shown to play a role in emotional processing [1], categorical indexing [2], as well as social learning [3]. Recently, unprecedented advancements have been achieved in the field of humanoid robotics. Modern robots are currently endowed with advanced systems that allow them to assume, amongst other things, different facial expressions and body postures, largely increasing their scientific interest and public popularity. This growing interest is reflected in a surge of research across diverse fields such as psychology [4], social robotics [5], and communication sciences [6], as well as extended to the real-world implementation of socially assistive robots for affective therapy, cognitive training, social facilitation, and even companionship (reviewed in [7]). In these cases, a robot’s ability to foster higher levels of communication and emotional resonance with human users is rooted in our ability to perceive relationships with non-human entities (like fictional characters or objects), especially in the absence of real interpersonal connections [8]. This phenomenon is largely driven by our tendency for anthropomorphizing, the cognitive process through which people attribute humanlike qualities to non-human agents [9,10]. As a result, robots that are designed with humanlike traits, specifically socially relevant traits like emotional faces and gestures, evoke a sense of familiarity in users, one that is believed to promote more naturalistic and emotionally resonant interactions [11]. Accordingly, robots with human-like facial features have emerged to assist individuals with a wide range of conditions affecting social information processing, including age-related cognitive decline [12], early life abuse [13], and stress-related disorders [14].

At this point, it has become relevant to examine how humans decode and understand robotic faces, to identify the neural correlates of robotic face processing, and to inform whether the mechanisms that support cognitive and emotional benefits in human–human interaction are effectively engaged for human–robot interactions. While prior studies have broadly explored this topic, it remains unclear whether robotic faces are processed similarly to humans, as the literature reports mixed results and methodological constraints related to differences in familiarity [15,16], anthropomorphism [17,18], and perceived agency [19] in different models of robot stimuli. Current results partially support the potential cognitive benefits of social robots, particularly in mental health contexts [20,21]. These research efforts are relevant to clarify the true benefits, appropriate applications, and limitations of social robots in supporting specialized human cognitive function [22].

Previous works examining evoked brain activity have characterized the temporal dynamics of human face processing, identifying key stages of perceptual encoding and categorization across different brain regions [23,24,25,26]. The N170 event-related potential (ERP) has emerged as a particularly important marker for face-sensitive information processing. It has been characterized as a cortical component occurring at approximately 130–200 ms in the form of a negative peak over the occipital-temporal areas of the brain following the presentation of faces, but not objects [27,28,29,30]. The N170 component has consistently been used as an electrophysiological marker reflecting the encoding of facial features, with current research exploring whether this component is modulated by emotional content or whether it is sensitive to humanoid facial structure alone [29]. This process is preceded by an early response to low-level visual features such as contrast, luminance, and spatial frequency manifested through a positive peak at around 80–120 ms post-stimulus onset called P100 [31,32].

Following the P100 and N170/vertex positive potential (VPP) complex, the later positive components are thought to reflect higher-order levels of facial information processing. The first of these, the P300, emerges around 250–500 ms and is primarily associated with attentional allocation and the evaluation of emotional or contextual significance [30,33,34]. While classically modulated by stimulus novelty in auditory oddball paradigms, recent work has demonstrated that the P300 amplitude is also sensitive to visual complexity [35], reflecting the increased attentional resources required to process intricate visual stimuli. The second, the P600, appears around 500–800 ms and is currently attributed to the re-evaluation and updating of gathered social and emotional information [36]. Usually elicited by syntactic errors and structural violations in language [37], recent findings indicate that this component reflects domain-general mechanisms of re-analysis and memory updating rather than purely linguistic processing [38]. Within this model, earlier components, namely the P100 and N170, mark the stage of structural encoding of facial information, while later P300 and P600 reflect higher-order cognitive processes involved in human face evaluations [39].

Recent research in related fields of Human–Computer Interaction (HCI) has increasingly relied on these electrophysiological markers to characterize cognitive responses to artificial agents. As reviewed by Kosch et al. [40], ERPs serve as robust, objective metrics for quantifying cognitive workload and attention allocation, independent of subjective reports. This methodology is critical for passive BCI systems, which utilize these components to monitor user engagement and detect perceptual mismatches during human–robot interaction [41]. Crucially, these distinctions extend to affective processing, where studies report that emotional avatars elicit unique ERP patterns compared to humans [42]. These results suggest the brain recruits distinct cortical networks when evaluating signals from artificial agents. Similarly, previous work has shown effects of task demands on specific face-sensitive ERP amplitudes, both in the processing of emotional human faces [43,44] and robotic faces [39,45]. However, despite these specific findings, how humans actively encode robotic faces has been examined to a lesser extent.

Here, we extend these results by examining how emotional content interacts with face categorization of human and robotic faces. Further, we address whether robotic faces effectively engage the same perceptual mechanisms as human faces and to what extent emotional expression modulates this activity. To do so, we examine brain responses to two different types of faces with distinct features through a simple categorization task, as it requires participants to actively discriminate between emotional expressions and thus ensures greater engagement with the perceptual cues of each face. During the task, electrocortical activity was recorded to investigate the effect of emotional facial expressions on robotic and human face perception by analyzing ERPs to each face category. We examined early and late evoked brain responses (over the P100, N170, P300, and P600 components) to happy and neutral human and robotic faces. We expected robotic faces to elicit enhanced early visual ERP waveforms, namely the P100 and N170 components, relative to the categorical decoding of faces. Differences in component amplitude at these stages would reflect the brain’s detection of both the structural similarity to human faces and their identity-related ambiguity. Further, we expected later components not to be affected by differences in face categories, as these features would already be resolved, but to show differences in emotional encoding of the faces, since these stages reflect a deeper evaluation of the emotional and social relevance of the stimulus [44].

Our results contribute to the growing body of knowledge on the neural mechanisms underpinning social cognition, face processing, and their applicability to emerging technologies such as humanoid robotics. As such, this research illustrates the neural substrates of face perception of biological and artificial entities and can also help inform the design and deployment of socially assistive robots, ensuring their effectiveness in applications with human users and establishing their potential to foster effective human–robot interactions.

## 2. Materials and Methods

### 2.1. Participants and Recruitment

A total of 46 participants (26 male, 20 female, n = 46) aged 18–27 years (M = 21.11, SD = 2.34) were recruited to take part in the experiment using online advertisement and word of mouth. Participants had no history of neurological disorders or substance abuse and had normal or corrected-to-normal vision. All subjects were provided with information about the nature of the experiment and gave their written informed consent prior to the beginning of the experiment session. The research protocol, including these data management and participant consent procedures, has been reviewed and approved by the ethics committees of Universidad Adolfo Ibáñez. All subjects received a small monetary compensation of approximately $20 USD for locomotion, time, and other expenses.

### 2.2. Procedure

Before the experimental task commenced, participants responded to a set of questionnaires to evaluate their attitudes towards robots, including measures of their perceived social influence, emotional influence, and situational interaction (see Section 2.5 for full details). During the experiment, participants sat approximately 60 cm from the computer screen in a quiet, dimly lit room. They were asked to maintain visual fixation at the center of the monitor throughout the task. Participants were instructed to categorize faces by emotion and to respond as quickly and accurately as possible using visual analog scales (VAS) and a computer keyboard [46]. This provided a simple yet effective measure of subjective preference and perception of emotional intensity. This continuous scale allows for greater sensitivity in detecting subtle differences in responses compared with categorical scales. A brief practice session consisting of a ten-picture trial was conducted to familiarize participants with the procedure, followed by two consecutive blocks of the main experimental task. The full set of 120 stimuli (detailed in Section 2.3) was presented in a pseudo-randomized order. Each face was shown exactly once, resulting in 30 realizations per condition (human–happy, human–neutral, robot–happy, robot–neutral) available for the grand average ERP analysis.

**Figure 1:**
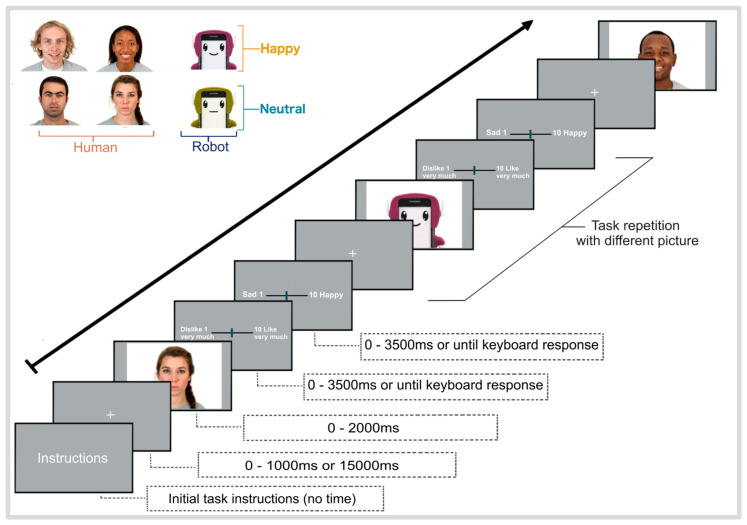
Schematic illustration of the stimulus presentation and visual analog scales for the testing sessions. Fixation crosses were presented with variable intervals to avoid anticipatory effects. Rating scales were presented in random order as well.

Each trial began with the presentation of a central fixation cross for approximately 1000 ms, with an added randomized time of ±500 ms in some of the pictures to prevent stimulus anticipation. Subsequently, a random face (either human or robot) was displayed for 2000 ms, followed by two VAS where participants rated the stimuli on likability (anchored with 1 dislike very much/10 like very much) and emotional valence perceived (anchored with 1 sad/10 happy). Participants had up to 3500 ms to respond using the keyboard; if no response was recorded within this time window, the trial proceeded automatically to the next one (Figure 1). Stimuli and rating scales were displayed on a PC monitor with an image resolution of 1707 × 1067 pixels (60 Hz monitor; frames updated every ~16.67 ms), using in-house Python v3.10 scripts. Randomly selected faces were controlled to avoid presenting the same experimental condition more than twice in a row.

### 2.3. Stimuli

A total of 120 color face images were employed in two experimental blocks. In total, 60 human face images (30 happy, 30 neutral) were chosen from the Chicago Face Dataset (CFD) [47]. Thirty female and thirty male face images depicted a frontal view against a white background. The remaining 60 robot face stimuli were created using images of a low-cost, readily available social robot (SimaRobot, [48]). These robot images were cropped and matched to the human faces image set for emotional expression: 30 pictures with hedonically positive “happy” expressions and 30 with “neutral” expressions (Figure 1). Robot stimuli were created using Krita v4.4 software, an open-source, free tool for image editing. Each image maintained identical lighting conditions (standardized diffuse illumination at a 45° angle), white background color, and viewing perspective (frontal orientation at 0° head rotation) to match their human counterparts. The distance and size of the agents were fixed so that the face occupied the same proportion of the visual field, and all images were set to 300 DPI. The four commercially available robot case colors were selected to be used as stimuli (magenta, red, blue, and green). Happy and neutral emotional expressions were selected because, unlike humans, most existing social assistive robots currently on the market are not designed to display negative emotions (e.g., anger, fear, or disgust), as seen in PARO [49], Pepper [50], NAO [51], Mabu [52], KOMPAÏ [53], and our SIMA robot. As such, only positive and neutral facial expressions were used.

### 2.4. EEG Recordings

Wireless EEG signals were recorded continuously at a sampling rate of 128 Hz using an Emotiv Epoc FLEX EEG gel-based system throughout the experimental task. This wireless EEG system comes with 32 sintered silver-silver chloride gel sensors (32 channels: AFz, FCz, Fp1, Fp2, F7, F3, Fz, F4, F8, FT9, FC5, FC1, FC2, FC6, FT10, T7, C3, Cz, C4, T8, CP5, CP1, CP2, CP6, TP9, TP10, P7, P3, Pz, P4, P8, O1, Oz, O2 + 2 references: CMS/DRL at TP9/TP10) and has been previously validated for visual components [54,55]. EEG sensors were placed in accordance with the international 10–20 system [56] and centered to the midpoint between anatomical landmarks on the scalp (nasion to inion and two identical preauricular sites). This system is a valuable and commercially affordable solution for EEG research. As is standard in this system, the analog signals were then digitized at 2048 Hz, filtered using a 5th-order sinc notch filter (50 Hz), and low-pass filtered before being down-sampled to 128 Hz [56]. The effective bandwidth of the EEG data was 0.16–43 Hz. The EEG cap was aligned with respect to the midpoint between the anatomical landmarks of the nasion and inion, and the left and right preauricular points. Electrode-to-skin impedances were lowered using electrolyte gel to a suitable level, as indicated by the “green light” in the EEG recording software EmotivPRO v3.4.1 [57], before starting the task.

### 2.5. Behavioral Measures

To investigate differences in participants’ preconceptions and attitudes that could influence their responses to robot stimuli, we administered the Negative Attitudes toward Robots Scale (NARS) [58] as a self-assessment tool. We used the Spanish version provided by Bartneck et al. (2005) [59], which was translated using a forward and backward translation process to ensure semantic accuracy. Attitudes are explored by inquiring within the scope of three sub-scales: Sub-scale 1, which explores negative attitudes toward situations and interactions with robots, Sub-scale 2, which explores negative attitudes toward social influence of robots, and lastly Sub-scale 3, which explores negative attitudes toward emotions in interaction with robots. Each sub-scale of the questionnaire gives a quantifiable measure of subjects’ predispositions to experience, discomfort, skepticism, or resistance that may emerge in different dimensions of human–robot interactions.

### 2.6. Electrophysiological Data Preprocessing

Raw EEG data were pre-processed in Matlab v2021b using the EEGLAB v2024.0 toolbox [60], the ERPLAB 10.0 toolbox [61], and automated with in-house scripts. Participants’ data were collected with good to average signal quality and were maintained throughout the sessions. The continuous data was high-pass filtered at 0.5 Hz with a 12 dB/oct roll-off. A working time window was selected from 2 s before the first stimulus to 2 s after the last one. Defective channels were initially identified by visual inspection and omitted from the preprocessing steps. Artifacts originating from eye blinks or movements, heartbeats, muscle contractions, channel noise, or electrical interference were identified and removed by means of independent component analysis (ICA) and using the ICLabel 1.4 classifier [62]; components with a score between 0.8 and 1 in the aforementioned artifactual categories were removed. At this point, any defective channels were automatically rejected using the CleanRawData v2.7 plugin set up with a 5 s flat line criterion, a 0.6 minimum channel correlation, and a kurtosis superior to five times the mean. Any remaining defective channels were spherically interpolated when possible. Data was then re-referenced to infinity with the REST 1.2 toolbox [63], low-pass filtered at 30 Hz with a 12 dB/oct roll-off, cut in (−200 ms 1000 ms) epochs around stimuli, and baseline-corrected relative to the mean voltage in the pre-stimulus time. Overall, participants retained a mean of 118.5 epochs (SD = 3.4), corresponding to 99% retention (SD = 2.2%). Per-condition retention ranged from 29.4 to 29.8 epochs (rejection rates 0.6–2.0%). Most participants retained all epochs or lost only one to two. Only two subjects reported higher rejection rates (Subject 16 = 4%; Subject 19 = 13%). However, their remaining data still achieved recommended thresholds for reliable ERP analyses.

### 2.7. ERP Analysis

Grand average ERPs were computed for human and robotic faces. A factorial 2 × 2 (human faces vs. robot faces, neutral vs. happy expression) ANOVA was used to compare waveform amplitudes at selected electrodes sites for the P100 (100–150 ms at electrodes O1, OZ and O2), N170 (150–200 ms at electrodes P7, P8, PO9, and PO10), P300 (290–350 ms at electrodes P3, Pz and P4), and P600 (600–800 ms at electrodes P3, Pz and P4) components.

Furthermore, evoked brain responses to robotic and human faces were examined using mass univariate analysis [64] performed on the Factorial Mass Univariate Toolbox (FMUT) [65] on Matlab. This approach was chosen to enable a fine-grained characterization of the temporal dynamics of neural activity at different experimental conditions across the whole scalp as well as to reduce potential error rates [66]. Factorial mass univariate analysis employing 10,000 permutations was performed across the entire epoch (0–990 ms) and at all electrode sites. FMUT works by conducting multiple ANOVAs throughout the ERP time series across the entire scalp instead of focusing on individual data points. Subsequently, it groups significant effects into clusters based on their timing and location over the scalp. FMUT then uses permutation testing to determine the significance of these clusters by comparing the observed cluster mass to a null distribution, ultimately calculating a *p*-value to assess statistical significance. To ensure full reproducibility of these results, the complete analysis code and detailed statistical outputs are available as Supplementary Materials at: https://rpubs.com/riverula/robot_2023 (accessed on 4 March 2025).

### 2.8. Behavioral Analysis

To examine any differences in rating behavior, response scores, and times, data were analyzed using Analysis of Variance (ANOVA) and paired *t*-tests on JASP v0.19.0 software [67]. Significant interactions were followed up using paired *t*-tests and considered significant at *p* < 0.05.

## 3. Results

### 3.1. Negative Attitudes Towards Robots (NARS) Scores

To investigate participants’ overall feelings regarding robots, three sub-scales of the NARS questionnaire, with a reported Cronbach’s alpha value from the original study of α = 0.80 [68], were used: Sub-scale 1, negative attitudes toward situations and interactions with robots (M = 13.33, SD = 3.30) (Cronbach’s α = 0.78); Sub-scale 2, negative attitudes toward social influence of robots (M = 14.78, SD = 3.21) (Cronbach’s α = 0.78); and Sub-scale 3, negative attitudes toward emotions in interaction with robots (M = 8.76, SD = 2.18) (Cronbach’s α = 0.65).

A significant main effect of sub-scale type on rating behavior was determined by one-way ANOVA (F(2, 144) = 55.90, *p* < 0.001). Tukey HSD post hoc comparisons indicated significant differences between sub-scale pairs (*p* < 0.01) wherein participants scored higher on S2 compared to Sub-scale 1 (MD = −1.45, SE = 0.59, *p* < 0.05), higher on Sub-scale 2 compared to Sub-scale 3 (MD = 6.02, SE = 0.59, *p* < 0.001), and significantly higher on Sub-scale 1 compared to Sub-scale 3 (MD = 4.57, SE = 0.59, *p* <0.001).

### 3.2. VAS Rating Scores

**Figure 2:**
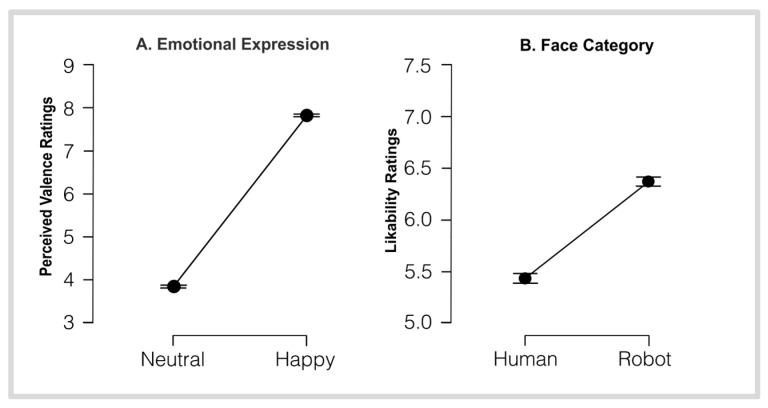
Line plots represent the distribution of rating scores for (**A**) emotional valence (1 sad/5 neutral/10 happy) and (**B**) likability (1 dislike very much/10 like very much). Error bars represent ±1 SE, with both comparisons showing a significant effect (*p* < 0.001).

A significant main effect of face category was observed on likability ratings, F(1, 18) = 225.24, *p* < 0.001, η^2^ = 0.04. Participants rated robotic faces (M = 6.37; SD = 2.17) as significantly more likable than human faces M = 5.43; SD = 2.30) (Figure 2B), t (5079.47) = −15.01, *p* < 0.001. Welch correction was applied due to the Brown-Forsythe test indicating violation of the assumption of equal variances. For emotional detection scores (Figure 2A), neutral expressions (M = 3.8; SD = 1.52) were rated significantly different from happy ones (M = 7.8; 1.78), t (df) = −85.61, *p* < 0.001.

Although “happy” faces were rated significantly higher than neutral ones (M = 7.8 vs. M = 3.8), ceiling scores were rarely reached. Participants distinguished the valence as positive, but the intensity was perceived as moderate-to-high rather than extreme, manifesting an accurate discrete emotion categorization independent of maximal ratings on continuous valence or arousal scales. Thus, the rating of 7.8 reflects a successful recognition of the emotion, scaled by the perceived intensity of the static stimuli.

### 3.3. ERP’s Results

**Figure 3:**
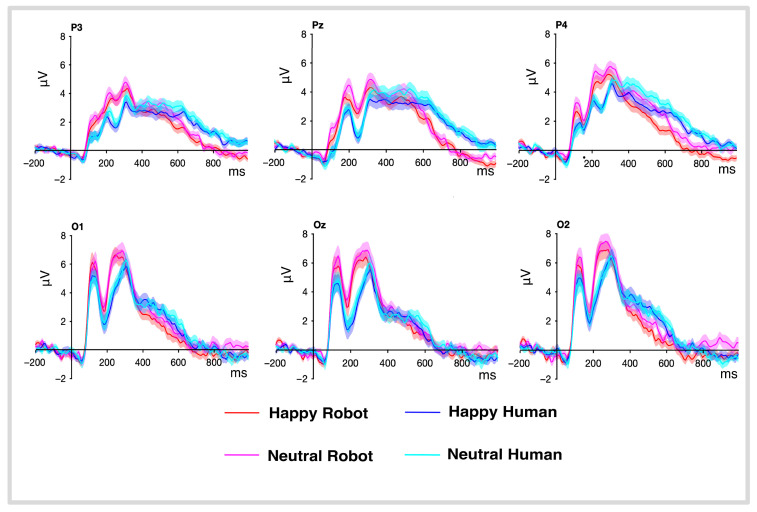
Group-averaged ERP waveforms in response to the different face categories and emotional valence visual stimuli, with shaded regions representing the standard error of the mean activity (μV) for each condition.

Grand average ERP waveform amplitudes were calculated for face category and emotional valence conditions. ERP component peaks were identifiable at 100 ms over occipital electrode sites and at 170 ms and 300 ms latencies over parietal electrode sites (Figure 3). These component peaks, as well as evoked activity at later periods (600 ms), were investigated.

#### 3.3.1. P100

**Figure 4:**
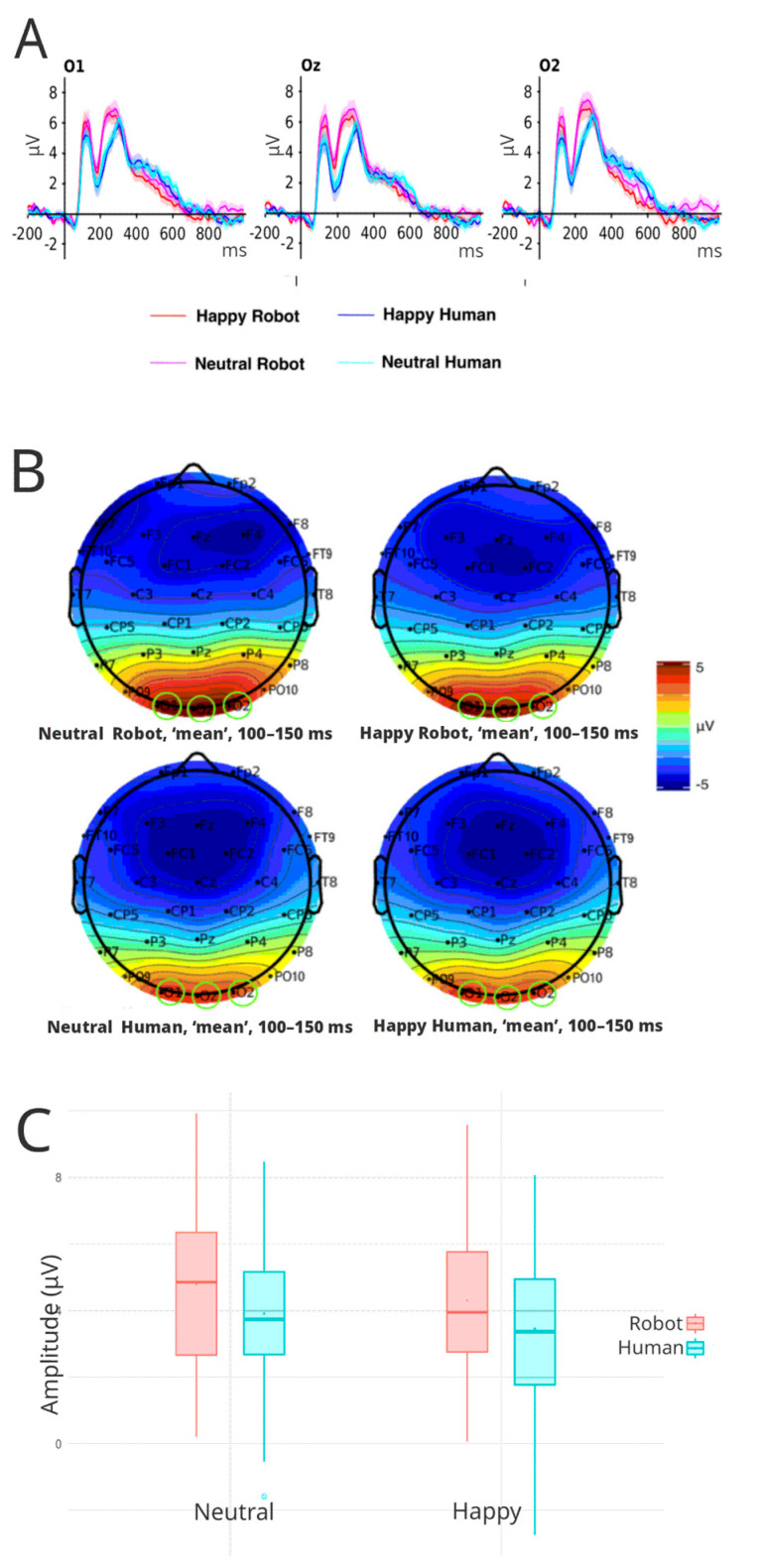
(**A**) Grand averaged P100 waveforms for all experimental conditions in each electrode of interest (O1, OZ, and O2). Red lines correspond to robotic faces, while blue lines correspond to human faces. (**B**) The average scalp voltage topography for each condition between 100 ms and 150 ms. Green circles identify electrodes of interest included in the analysis. (**C**) Boxplots of average amplitude (μV) for happy and neutral expressions in human and robot face categories over all electrodes of interest (O1, Oz, O2).

Our analysis revealed a significant effect of face type on amplitude differences in P100 (F(1, 45) = 13.50, *p* < 0.001, η^2^ = 0.021). To gain a more detailed understanding of how each factor (Figure 4A) influences individual ERP amplitudes, mean estimates and contrast analyses were performed for each significant factor. These analyses showed that robotic faces elicited greater amplitudes ($\overset{-}{x}$ = 5.59 μV, 95% CI: 4.44–6.75) compared to human faces ($\overset{-}{x}$ = 4.52 μV, 95% CI: 4.66–5.37) (t (45) = 3.67, *p* < 0.001) (Figure 4B). Multiple comparisons were corrected using the Greenhouse–Geisser (GG) method. No effect was found in the emotional valence condition.

#### 3.3.2. N170

**Figure 5:**
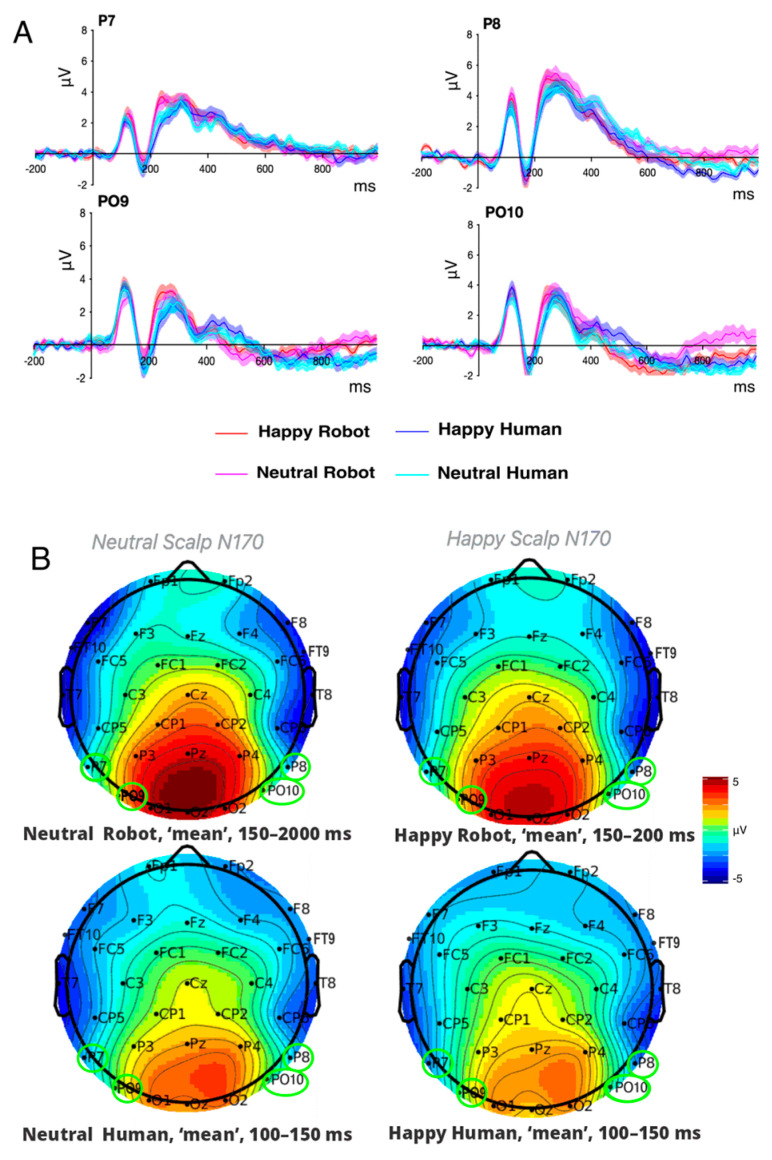
(**A**) Grand average ERP waveforms for the N170 component across different face categories and emotional valence conditions, at selected electrodes P7, P8, PO9, and PO10. (**B**) N170 scalp map topographical plots display the average scalp voltage distribution for each condition within the 150 to 200 ms time window. In the same way as in the previous component, no effect was found in the emotional valence condition. Green circles indicate selected electrodes for analysis.

No main effect of face category on amplitude differences was found in our analysis (Figure 5A). However, a significant interaction between face and medio-lateral scalp position (P3, P4) was observed (F(1, 45) = 6.34, *p* = 0.015, η^2^ = 0.002). Follow-up comparisons revealed a significant difference at the left medio-lateral sites (t (45) = 2.67, *p* = 0.049), with robot faces eliciting higher amplitude at left positions ($\overset{-}{x}$ = 0.16 μV, IC 95%: −0.57–0.90) than human faces ($\overset{-}{x}$ = −0.37 μV, IC 95%: −1.10–0.36) (Figure 5B). No further effects relating to valence or electrode position were observed.

#### 3.3.3. P300

**Figure 6:**
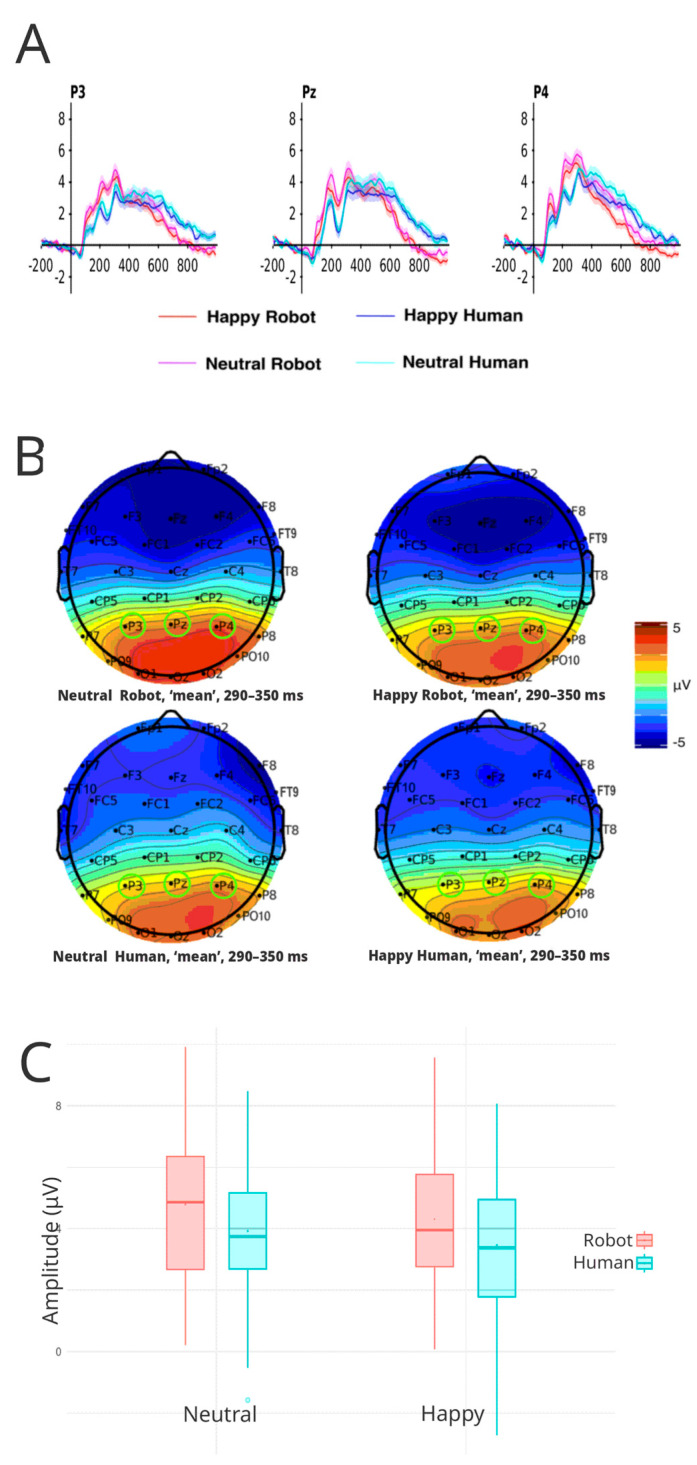
(**A**) The P300 waveforms for all experimental conditions in each electrode of interest (P3, Pz, and P4). Red lines correspond to robotic faces, while blue lines correspond to human faces. (**B**) The average scalp voltage topography for each condition between 290 ms and 350 ms. Green circles identify electrodes of interest included in the analysis. (**C**) Boxplots of average amplitude (μV) for happy and neutral expressions in human and robot face categories over all electrodes of interest (P3, Pz, and P4).

The ANOVA showed a significant effect in amplitude differences for face category (F(1, 45) = 10.59, *p* = 0.002, η^2^ = 0.028). Similarly, the emotional valence (happy vs. neutral) also displayed a significant effect on the amplitude of the P3 component (F(1, 45) = 7.80, *p* = 0.008, η^2^ = 0.008) as well as electrode site (F(1.86, 83.63) = 11.13, *p* < 0.001, η^2^ = 0.030), showing significant differences in amplitude, specifically between the electrodes P3–P4 (t (45) = −4.50, *p* < 0.001) and between the electrodes Pz–P4 (t (45) = −3.11, *p* < 0.01) were found (Figure 6A).

Analysis of the mean amplitude of each face category showed robot faces ($\overset{-}{x}$ = 4.55 μV; IC 95%: 3.90–5.19) elicited stronger P300 amplitudes than human faces ($\overset{-}{x}$ = 3.69 μV; IC 95%: 3.05–4.33) (t (45) = 3.22, *p* = 0.002) in favor of robot faces (Figure 6B). Further analysis on the effect of valence revealed that happy faces ($\overset{-}{x}$ = 3.88 μV; IC 95%: 3.29–4.48) evoked weaker amplitudes than neutral faces ($\overset{-}{x}$ = 4.35 μV; IC 95%: 3.73–4.98) (t (45) = 2.79, *p* = 0.007).

Lastly, analysis of the electrode factor revealed the following average amplitudes: P3 (M = 3.69 μV, 95% CI: 3.10–4.28), Pz (M = 3.93 μV, 95% CI: 3.20–4.66), and P4 (M = 4.74 μV, 95% CI: 4.14–5.33). The results indicate significant variations in cortical responses, specifically between P3 and P4 locations (t (45) = −4.50, *p* = 0.0001) and between Pz and P4 (t (45) = −3.11, *p* = 0.008). Multiple comparisons were corrected using the Greenhouse-Geisser (GG) method.

#### 3.3.4. P600

**Figure 7:**
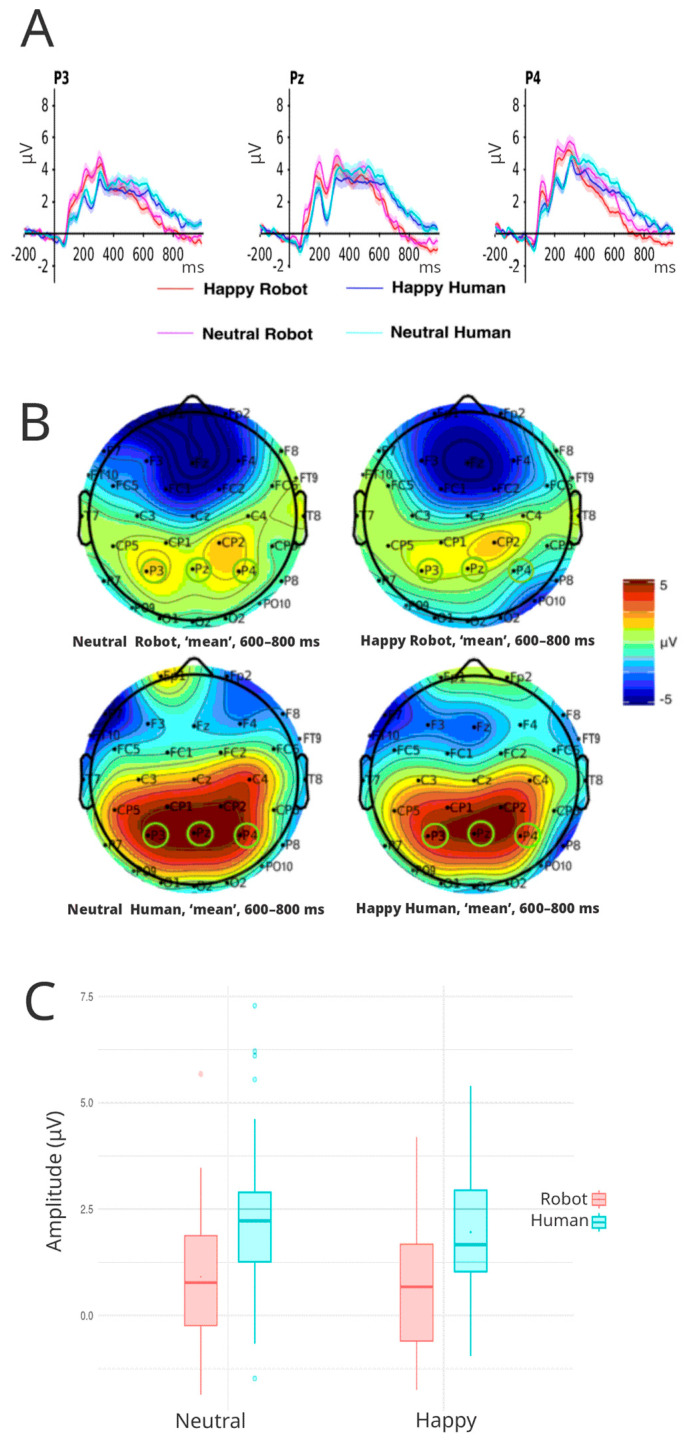
(**A**) P600 waveforms for all experimental conditions in each electrode of interest (P3, Pz, and P4). Red lines correspond to robotic faces, while blue lines correspond to human faces. (**B**) Shows the average scalp voltage topography for each condition between 600 ms and 800 ms. Green circles identify electrodes of interest included in the analysis. (**C**) Boxplots of average amplitude (μV) for happy and neutral expressions in human and robot face categories over all electrodes of interest (P3, Pz, and P4).

The EEG analysis identified a notable late component potential occurring between 600 and 800 ms, in which further analysis showed a significant effect of face category; F(1, 45) = 54.35, *p* < 0.001, η^2^ = 0.128 (Figure 7A). Robot faces showed a mean P600 amplitude of 0.79 μV (IC 95%: 0.38–1.21), while human faces showed a significantly higher mean amplitude of 2.09 μV (IC 95%: 1.67–2.51) (t (45) = −7.37, *p* = 0.0001).

The interaction between face type and electrode site also showed a significant effect of F(1.99, 89.60) = 3.18, *p* = 0.046, η^2^ = 0.002, showing higher mean amplitudes for the human condition on the electrodes P3: M = 2.02 μV (95% CI: 1.61–2.44), Pz: M = 2.30 μV (95% CI: 1.79–2.81) and P4: M = 1.95 μV (95% CI: 1.53–2.37) than the robot condition in the electrodes P3: M = 0.91 μV (95% CI: 0.50–1.32), Pz: M = 0.79 μV (95% CI: 0.33–1.26) and P4: M = 0.67 μV (95% CI: 0.23–1.12) (Figure 7B). Contrast calculations found a statistically significant contrast just for human faces, found between Pz and P4: (t (45) = 2.54, *p* = 0.038), indicating a higher mean amplitude at electrode Pz than at electrode P4.

Ultimately, interactions between face valence and electrode also manifested statistically significant differences, F(1.86, 83.87) = 3.91, *p* = 0.026, η^2^ = 0.002, showing higher mean amplitudes for neutral faces on the electrodes P3: M = 1.58 μV (95% CI: 1.17–2.00), Pz: M = 1.59 μV (95% CI: 1.09–2.09) and P4: M = 1.53 μV (95% CI: 1.12–1.94) than for happy faces in the electrodes P3: M = 1.35 μV (95% CI: 0.93–1.77), Pz: M = 1.51 μV (95% CI: 1.07–1.95), and P4: M = 1.10 μV (95% CI: 0.71–1.49). Happy faces contrast analysis revealed a statistically significant difference (t (45) = 3.35, *p* = 0.004) only between electrodes Pz and P4.

#### 3.3.5. ERP’s Summary

A summary of the statistical effects for all ERP components is provided in Table 1. As shown, the effect of face category exerted a significant influence across all time windows, though the direction of the effect shifted over time. Specifically, robot faces elicited significantly larger amplitudes than human faces during the early and mid-latency stages (P100, N170, and P300). However, this pattern reversed in the late-latency stage, with the P600 component showing significantly larger amplitudes for human faces compared to robot faces (*p* < 0.001). Similarly, valence showed no significant effects on early sensory components (P100, N170) present in our data. Significant effects, however, emerged in the later cognitive components (P300 and P600), where neutral faces consistently elicited larger amplitudes than happy faces, regardless of the agent type. For a complete statistical overview, please refer to Table S1 in the Supplementary Materials.

### 3.4. Factorial Mass Univariate Analysis Results

**Figure 8:**
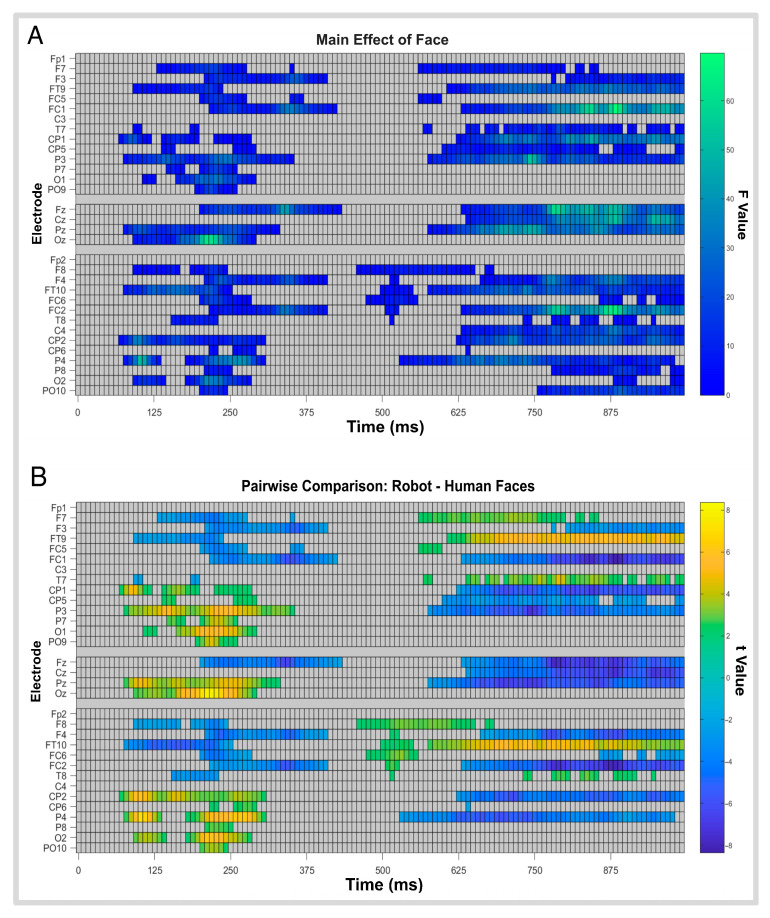
(**A**) Main effect of face across conditions (human and robot). Rectangles indicate electrodes and time points with statistically significant differences in brain activity as measured by F-values; warmer colors indicate stronger effects; gray areas indicate non-significant points. (**B**) Pairwise comparison of robot minus human faces. Rectangles indicate electrodes/time points with significant t-values; darker colors indicate bigger differences, lighter colors indicate weaker differences, and gray areas indicate non-significant points.

A mass univariate permutation analysis was performed at the intra-subject level to analyze the sensitivity of the electroencephalographic data in distinguishing between face and emotion categories (Figure 8). Firstly, the analysis effectively ruled out any significant interaction effects between emotional and face categories. An analysis of each individual variable revealed statistically significant cluster formation in the presence of face stimuli over centro-occipital and right parietal sites (Figure 8A). Additional clusters were observed at early temporal stages around 100 to 400 ms, which were predominant in centro-medial sites, and at later stages, between 500 and 990 ms, mainly over left and right fronto-central, parietal, and centro-medial regions.

Further analysis of voltage characteristic effects of robots relative to human faces (Figure 8B) revealed a predominance of higher voltage clusters for human faces relative to robot ones from around 100 ms to 375 ms in electrodes [F7-F3-FT9-FC5-FC1], [Fz], and [F8-F4-FT10-FC6-FC2-T8], and late stages from around 500 ms to 990 ms at electrodes [FC1, CP1-CP5-P3], [FZ-CZ-PZ], [F3], [FC2], [CP2], and [P4].

These effects were guided by differences brought about by human faces seen over early stages from around 100 ms to 375 ms in left and right centro-parietal and occipital sites, at electrodes [CP1-CP5-P3-P7-O1-PO9] and [CP2-CP6-P4-P8-O2-PO10], and late stages (around 500–990 ms) in frontal and frontal-temporal sites, at electrodes [F7-FT9-T7] and [F8-FT10].

**Figure 9:**
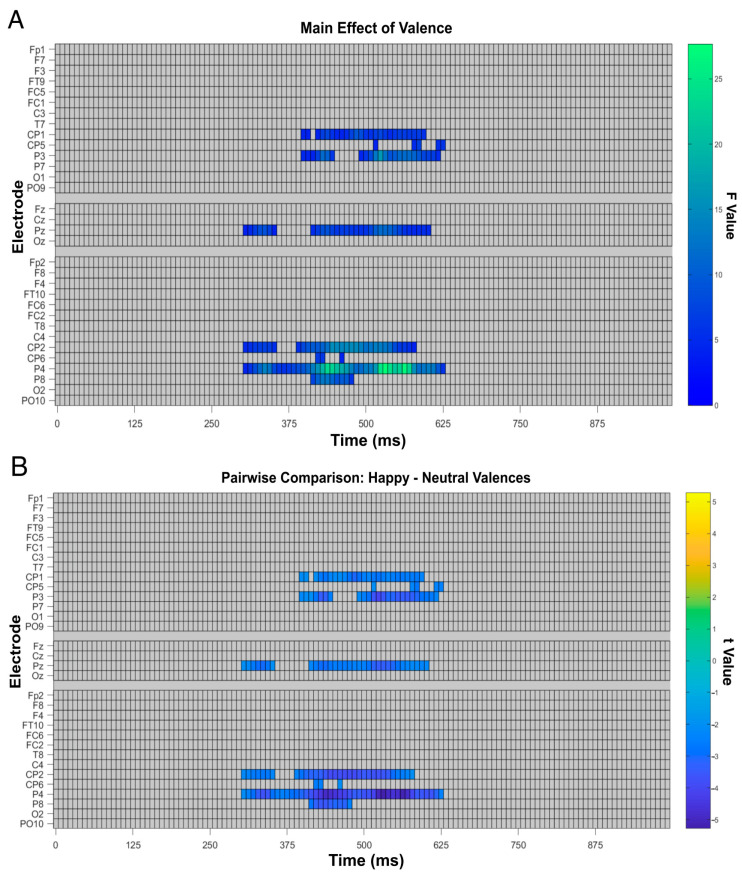
ANOVA task effect for: (**A**) FMUT valence raster plot where f-score values are represented by warmer colors, which correspond to stronger effects, while gray rectangles indicate areas with no statistical significance; and (**B**) mass valence comparison raster plot of t-score values are represented by warmer colors, which correspond to stronger effects, while gray rectangles indicate areas with no statistical significance.

Figure 9A depicts valence-related effects on waveform amplitude for face valence, where significant clusters can be found around 300–625 ms situated at left and right centro-parietal and parietal electrodes [CP1-CP5-P3], [CP2-CP6-P4-P8], as well as the centro-medial electrode [Pz]. Further analysis of its voltage characteristics shows no differences (Figure 9B). Valence-related clusters not only do not overlap with previous face category clusters but also appear to encode these differences at latencies where face categories do not.

## 4. Discussion

Our results show specialized human face-processing mechanisms engage differently with artificial faces. Robotic faces elicited enhanced early and mid-latency brain responses (P100–N170-P300), reflecting category-specific effects at the structural-encoding stage. Emotional expression, in turn, modulated both mid-latency (P300) and late-stage (P600) activity. Moreover, face category effects persisted into later stages, P300 and P600, suggesting that category distinctions extend into higher-order processing as well.

In contrast to previous studies where robotic faces did not seem to modulate early stages of face information processing [44,69], here, early P100 and N170 functional differences were significantly influenced by face category, characterized by enhanced amplitudes evoked by robot faces over human ones. Since early components index sensory processing and attentional allocation [70], these effects can be partially explained by two factors known to modulate such processes: perceptual novelty and expertise. Relative expertise enables faster and optimized perceptual processing of a specific category, resulting in reduced component latency and amplitude [70,71]. This reduction reflects a more economical use of cognitive resources, which may explain the attenuated responses to human faces due to greater familiarity. In contrast, robotic faces are both less familiar and visually informative than human faces in terms of low-level properties [44,72]. Robotic faces such as the SIMA robot used here contain fewer low-level visual cues, such as contrast, luminosity, shapes, and textures. Fewer diagnostic cues intrinsically make robotic faces less predictable to the observer, rendering them more novel and requiring additional attentional and perceptual resources to interpret. Therefore, robotic faces with heightened amplitudes would reflect the greater cognitive effort needed to process less informative and unfamiliar visual features [73].

Differences between robots and human faces were also found to modulate the lateralization of the N170 component. As interaction effects indicate, effective discrimination happened particularly in the left hemisphere, suggesting that even when the right hemisphere classically has been established as dominating the face processing of human faces [74,75], the left hemisphere may play a key role in the differential processing of human and robot faces at the N170 latency. Furthermore, the absence of emotional effects on early components’ amplitudes aligns with the literature showing that emotion is encoded at later stages of face processing. This finding further clarifies the role of the N170 component in encoding emotional information of faces, with dual evidence of both task insensitivity and modulation [76]. These findings align with previous dual-process theories positing that structural encoding of facial features, specifically facial configuration rather than emotional valence, occurs at this stage [45,77].

Beyond early perceptual responses, the analysis of mid- to late-stage ERP components revealed systematic effects of both face category and emotional valence. Big differences for emotional expressions, with higher amplitudes observed for neutral faces likely reflecting enhanced attentional allocation and stimulus evaluation of top-down distinctions, especially in neutral expressions, which are inherently ambiguous and thus demand greater processing resources to assess their social relevance [30]. More importantly, the modulation of mid-component P300 by face category suggests that robotic faces have not been fully resolved as “face-like” early on, triggering continued scrutiny and incomplete integration into social-cognitive representations. However, this effect does not extend into later stages.

The latter stages encompassing the P600 component latency have been previously associated with syntactic reanalysis and structural reencoding during cognitive tasks [78]. As such, enhanced P600 amplitudes to human faces would likely reflect greater cognitive effort for structural reanalysis or updating of social and emotional cues, which are less prominent in robotic faces. This interpretation aligns with proposals that the P600 indexes the re-evaluation and updating of mental representations [79] and is further supported by the evolutionary salience of human faces. These results contribute to clarifying the unresolved functional role of the P600 in face processing [80] and furthering our current understanding of robotic face processing. Simultaneously, the functional profile of this effect partly overlaps with the Late Positive Potential (LPP), as shown in our whole scalp analysis. The LPP is well documented for its sensitivity to semantic and emotional salience and is characterized as a marker of higher-order evaluation, categorization, top-down processing, and sustained attention allocation [81,82,83,84]. Importantly, its amplitude is reported to scale with facial realism and human-likeness [85,86]. This aligns with our finding of stronger responses to humans compared to robotic faces, which allude to these differences reflecting evaluative and attentional processes that are engaged when faces are biologically salient and socially significant.

Extending this functional framework to applied domains, the progression from P300-indexed scrutiny reflecting incomplete social-cognitive integration to P600-mediated re-evaluation supports a unified interpretation of these components as neural correlates of an “exhaustive search” mechanism [87]. Under this framework, the sustained late positivity signifies the recruitment of additional neural resources necessitated by the ambiguity of synthetic stimuli. Unlike the efficient, self-terminating processing often observed for biological faces, this obligatory search for categorization imposes a distinct cognitive cost. In the context of Human–Computer Interaction (HCI), such increased workload disrupts the fluidity of interaction, potentially precipitating cognitive fatigue [88] or even the psychophysiological unease associated with the “uncanny valley” [89]. Consequently, these mid- to late-latency ERPs possess significant translational utility for adaptive Brain–Computer Interface (BCI) systems. By leveraging these indices of exhaustive search as real-time neuro-markers, future synthetic agents could autonomously detect user cognitive dissonance and dynamically calibrate their behavior to ensure smoother, more naturalistic integration.

This dynamic shift across processing stages is mirrored in our behavioral data, where higher robot likability ratings reflect the immediate positive appeal of robot faces (Figure 2B); meanwhile, NARS’ prominent score on sub-scales Sub-scale 2 and Sub-scale 1 indicates later evaluative caution about their social-interactive competence. These patterns add depth to the ERP results by linking neural dynamics to the temporal unfolding of participants’ dual attitudinal responses. Furthermore, FMUT whole scalp analysis revealed simultaneous dual processing across two distinct temporal phases corresponding to separate stages of information processing and decoding, resulting in distinct activation patterns and encoding strategies when processing different face types or emotional valences. Consequently, early P100 and N170 to P600 and further time windows show heightened activation when processing different face types, while the P300 and P600 time windows show significant activations when decoding emotional valence. This inversion provides further insight into differentiation in the neural coding of faces, where the processing of different face types and emotional content involves distinct neural coding stages at a temporal and spatial distribution level. The bilateral activity patterns shown here are consistent with prior neuroimaging studies on face perception [90,91]. This bilateral engagement supports the view, aligned with the N170 discussion, that face processing is distributed across both hemispheres rather than confined to one [75].

While the study provides valuable insights, certain limitations must be acknowledged. One limitation involves the stimulus design and the absence of negative robot expressions, which may constrain interpretation, as negative human expressions are known to elicit distinct temporal and spatial ERP dynamics due to their evolutionary relevance and strong affective resonance. Future research should address this gap by incorporating a broader repertoire of ecologically plausible negative robotic expressions as they become available. Additionally, the study did not include a non-face control condition, which could clarify whether ERP modulations such as P1 reflect face-specific or general visual processing. Furthermore, the precise association between P100 modulation and cognitive workload remains insufficiently understood, limiting the interpretative power of early visual ERP findings and warranting further investigation. One additional limitation concerns the analytical approach. Traditional ERP analyses focus on predefined electrodes and time windows, potentially overlooking distributed effects [34,64,66]. Mass univariate analyses were employed to allow data-driven examination across all electrodes and time points, increasing sensitivity to subtle effects of face type and emotional valence. However, reconciling the interpretability of component-based and exploratory approaches remains unresolved, and future work should develop integrative frameworks to capture overlapping spatio-temporal dynamics.

## 5. Conclusions

This study provides electrophysiological evidence for distinct time courses in the processing of human versus robotic facial expressions, demonstrating that robotic faces capture early perceptual attention and interest, whereas human faces retain higher-order socio-emotional salience. Specifically, robotic faces elicit closer perceptual attention during early visual processing (P100), likely due to their visual novelty. Furthermore, although robotic stimuli successfully recruit face-specific structural encoding mechanisms (N170) despite their artificial nature, this engagement is less robust compared to human faces. Consequently, robotic faces require re-evaluation at the mid-latency stages (P300 and P600), reflecting their incomplete integration into social-cognitive representations. Notably, humans do not significantly categorize or process emotional expressions from robotic faces at early key time stages (P100 and N170).

These findings are significant for the employment of robots, as the inability to autonomously detect emotional valences and process robotic emotional expressions could hinder their effectiveness in therapeutic and social settings, especially when emotion recognition is crucial for fostering meaningful human–robot interactions. As such, future clinical research should examine socio-emotional effects in early components and face-specific brain responses, employing stronger methodological rigor and greater diversity in study conditions, to better understand the variability in outcomes and develop more accurate models of human–robot neurophysiological data.

## Figures and Tables

**Table 1:** Summarizes the statistical effects for averaged ERP amplitudes. The table presents the main effects of face category and valence across the P100, N170, P300, and P600 components. Significant F-values, *p*-values, and effect sizes (η^2^) are reported as well as the direction of the effect.

ERP					
Component	Factor	Direction of Effect	Test Statistic	*p*	η^2^
P100	Face Category	Robot > Human	*F*(1, 45) = 13.50	<0.001	0.021
	Valence	—	ns	ns	ns
N170	Face Category	Robot > Human (Left Sites) ^a^	*F*(1, 45) = 6.34	0.015	0.002
	Valence	—	ns	ns	ns
P300	Face Category	Robot > Human	*F*(1, 45) = 10.59	0.002	0.028
	Valence	Neutral > Happy	*F*(1, 45) = 7.80	0.008	0.008
P600	Face Category	Human > Robot	*F*(1, 45) = 54.35	<0.001	0.128
	Valence	Neutral > Happy ^b^	*F*(1.86, 83.87) = 3.91	0.026	0.002

Note. ns = non-significant. η^2^ = eta squared. ^a^ Interaction between Face and Scalp Position. ^b^ Interaction between Valence and Electrode Site.

## Data Availability

The original contributions presented in the study are included in the article, and original data can be found online Angélica, Xaviera; Soto, Vicente (2025), “M_Robot”, Mendeley Data, V1, doi: 10.17632/7rydn8vpnd.1 (https://data.mendeley.com/preview/7rydn8vpnd?a=621faa7c-de74-4370-bd82-548c61fe0efa (accessed on 16 December 2025)). further inquiries or requests can be directed to the corresponding author.

## Supplementary Materials

The following supporting information can be downloaded at: https://rpubs.com/riverula/robot_2023 (accessed on 4 March 2025), Figures: ERP plots, topographic maps, voltage distributions, and ANOVA results for P1, P3, and late components. Tables: Summary statistics, ANOVA tables, and means contrasts. Code: Preprocessing and analysis scripts for MATLAB 2019b (EEGLAB 2024.0, ERPLAB 10.0, and custom scripts). References [60,61,62,63,92,93,94,95] are cited in Supplementary Materials.

## Abbreviations

The following abbreviations are used in this manuscript:

EEG	Electroencephalography
ERP	Event-related potential
FMUT	Functional mass univariate analysis toolbox
NARS	Negative Attitudes Towards Robots Scale
VPP	Vertex positive potential
VAS	Visual analog scale
ICA	Independent Component Analysis
ANOVA	Analysis of Variance
